# Fistula of the Stomach

**Published:** 1833

**Authors:** John H. Cook

**Affiliations:** Pendleton, Madison Co., Indiana


### Art. IV.—Fistula of the Stomach.

*Notes of a case of Fistulous opening of the Stomach, successfully treated, by* Dr. John H. Cook.

Sometime in the month of February, 1832, Dr. Bardwell and myself were called to visit the widow D., aged 39 years. We found her, as near as may be, in the following condition: A fistulous opening, immediately by the side of the umbilicus, into which a buck shot might have been readily passed; on removing the bandage, a gill of bile was suddenly discharged; after which, a small quantity of a different (the gastric?) fluid, came slowly away. These discharges were attended with great pain, on account of their acrid quality. The whole surface of the abdomen was excoriated, inflamed and intolerably painful. We introduced a flexible catheter, its whole length, 13 inches, before meeting with any resistance, when the extremity suddenly met with an obstacle. By pushing it against the resisting body, or even by slightly agitating the instrument, strong efforts to vomit were produced.

Withdrawing the catheter, we desired her to drink a glass of water; she did so, and in 20 seconds, the *whole* was discharged through the fistula, as we ascertained, by measuring it. The direction of the fistula was upward, and slightly inclining backwards, with about the same inclination to the right side. We came to the conclusion, that the opening within, was at, or about, the pyloric orifice of the stomach; and that the catheter entered the stomach, and pressed against its cardial portion. The patient even felt it there, and applied her hand externally over that part.

### Treatment.

Taking a large beef's bladder, we cut it open, longitudinally, spread it well with adhesive plaster, and after washing the inflamed surface, and dressing it with basilicon spread on fine linen, we applied the bladder over the abdomen, and made an opening over the fistula, through which the matter might escape. We then applied a bandage and compress, and directed that it should be reapplied immediately after each discharge. We advised mucilaginous drinks, and a diet of rye mush and molasses, and nourishing enemata. The patient was much emaciated for want of proper nourishment, as every thing passed off undigested through the fistula. No evacuation had taken place in the natural way, for ten days previous to our visit. The external irritation of the abdomen soon healed, and the bladder was then applied to the skin as a protection, and continued there with the happiest effect. The bandage was gradually tightened, and a compress of a cylindrical form was laid over the course of the fistulous canal. By these means our patient regularly, but slowly recovered. In a few days the alvine evacuations were restored to their natural outlet, and the discharges from the fistula began to decrease. In 30 days the opening was closed, and the fistula apparently obliterated. Several months have elapsed since that event, and she continues in excellent health.

All we could collect from her as to the history of her case, was this: Six months before, in one of the south-east counties of this State, she was attacked with constipation and violent pain at the pit of the stomach, which resisted every remedy, till the 19th day, when the fistula showed itself.

*Pendleton, Madison Co., Indiana, Nov.* 1833.

